# Evaluation of the microencapsulation of orange essential oil in biopolymers by using a spray-drying process

**DOI:** 10.1038/s41598-020-68823-4

**Published:** 2020-07-16

**Authors:** Maria Clara Santana Aguiar, Maria Fátima das Graças Fernandes da Silva, João Batista Fernandes, Moacir Rossi Forim

**Affiliations:** Department of Chemistry, Federal University of São Carlos, Rod. Washington Luiz, Km 235, São Carlos, SP 13565-905 Brazil

**Keywords:** Biomaterials, Drug delivery, Chemistry, Analytical chemistry, Materials chemistry

## Abstract

Essential oils are volatile compounds commonly used by several industries, easily degradable, which restrains their applications. Therefore, we developed and validated a methodology for producing microcapsules loaded with orange essential oil, using a spray-drying process. The experimental design results showed that the combination between a low flow transfer rate (0.15 L h^−1^) of the colloidal suspension, a higher drying air flow rate (536 L h^−1^), and an inlet air temperature of 150 °C to the spray-dryer were the most important parameters for the atomization efficiency. The method optimization resulted in microcapsules with powder recovery between 7.6 and 79.9% (w w^−1^), oil content ranging from 8.9 to 90.4% (w w^−1^), encapsulation efficiency between 5.7 and 97.0% (w w^−1^), and particle sizes with a high frequency of distribution less than 4 μm. In these experiments, gelatin and lignin were evaluated as biopolymers of encapsulation. We also developed an analytical method using headspace gas chromatography. The matrix effects could be addressed by using matrix-matched calibration curves. The chromatographic analysis was linear and selective for d-limonene between 0.025 and 3.00 µg mL^−1^, with correlation coefficients higher than 0.99. The analytical method had limits of detection and quantitation of 0.024 and 0.073 mg g^−1^ for gelatin and 0.039 and 0.119 mg g^−1^ for lignin, respectively.

## Introduction

Essential oils (EOs) are botanical secondary metabolites of low molecular mass^1^. These compounds have gained considerable attention because of their applications in cosmetics, detergents, medicines, food production, and pest protection^2,3^. Among EOs, orange essential oil stands out since it is an abundant coproduct from citrus processing that has a characteristic flavor and fragrance and has antimicrobial, antifungal, and antioxidant activities^4–6^.

However, few EOs are water-soluble and they are easily degraded by heat, oxidation, and light^7^. To improve their dispersion in an aqueous medium, biological action, and protection from environmental agents, methodologies that promote the encapsulation of EOs have been developed^7–9^. Among the methods for encapsulating essential oils, spray-drying has received attention because of its low operational cost, compatibility with labile materials, it can produce stable final products, and compatibility with continuous large-scale production^10,11^.

Because of the multiple variables that can influence the spray-drying process, the use of biopolymers as wall material is a research area of increasing interest. In this group, gelatin and lignin can be ideal candidates. In addition to their structural characteristics and ability to self-associate^10,12^, these products are biodegradable and biocompatible materials^12,13^. Moreover, it has a low cost that is favorable from a commercial point of view. The lignin is a waste product of the pulp and paper industry.

Some studies have been conducted to evaluate the encapsulation of orange essential oil using spray-drying process. In these studies, a variety of polymers such as modified starch, maltodextrin, whey protein, cellulose nanofibrils, and Arabic gum were reported as showing potential for application in atomization drying process^14–16^. However, there is little information available on the encapsulation of orange essential oil using gelatin or lignin as biopolymers.

Despite the promising data, there is a lack of adequate information in the literature that not only describes validated methodologies for the encapsulation of essential oils but also describes reliable analytical methods that meet the requirements of analytical applications, especially for the analysis of atomization efficiency. Quantitative analysis in quality control of the formulated essential oil is important in determining the features of the final product (reproducibility, stability, dispersion, release kinetics, etc.), as well as its relationship to the desired biological activities.

Considering the need to develop methods for the encapsulation of EOs and quality control analyses, the main aims of this present work were to propose a method using spray-drying for the encapsulation of orange essential oil and its subsequent quality control analysis through quantification by Headspace Gas Chromatography with Flame Ionization Detector (HS-GC-FID). Moreover, the developed analytical method was validated through different parameters, and the essential oil was characterized by gas chromatography-mass spectrometry.

## Results and discussion

### Matrix characterization

The volatile constituents of the orange EO, their retention time, and the relative area corresponding to each compound are described in Supplementary Table S1. In this analysis, we verified the presence of 27 different compounds and identified 24. We chose to highlight d-limonene (77.5%), β-myrcene (11.1%), α-pinene (3.99%) and linalool (1.44%) because these four constituents represented 94% of the total relative abundance area. These terpenes were also found to be the major constituents when orange essential oil was extracted by a Clevenger apparatus^4^, supercritical fluid^17^, and microwave steam distillation^18^, and they were related to the biological activity of orange EO. Since *d*-limonene was the major component identified, we selected it as a quality control marker for the development of an analytical method to be used to determine the atomization efficiency.

### Development of orange essential oil encapsulation methodology

One of the great difficulties in working with essential oils is to prevent the loss of its volatile constituents. Moreover, for microencapsulated products loaded with essential oil, we also face problems of the compatibility of the polymers with the chromatographic analyses. In most nano- and microencapsulation protocols such as spray-drying, heating steps can result in the partial loss of the essential oil, resulting in both economical losses and difficulties in interpreting the results of the biological assays. In this context, the optimization and analysis of EO content are critical.

**Figure 1:**
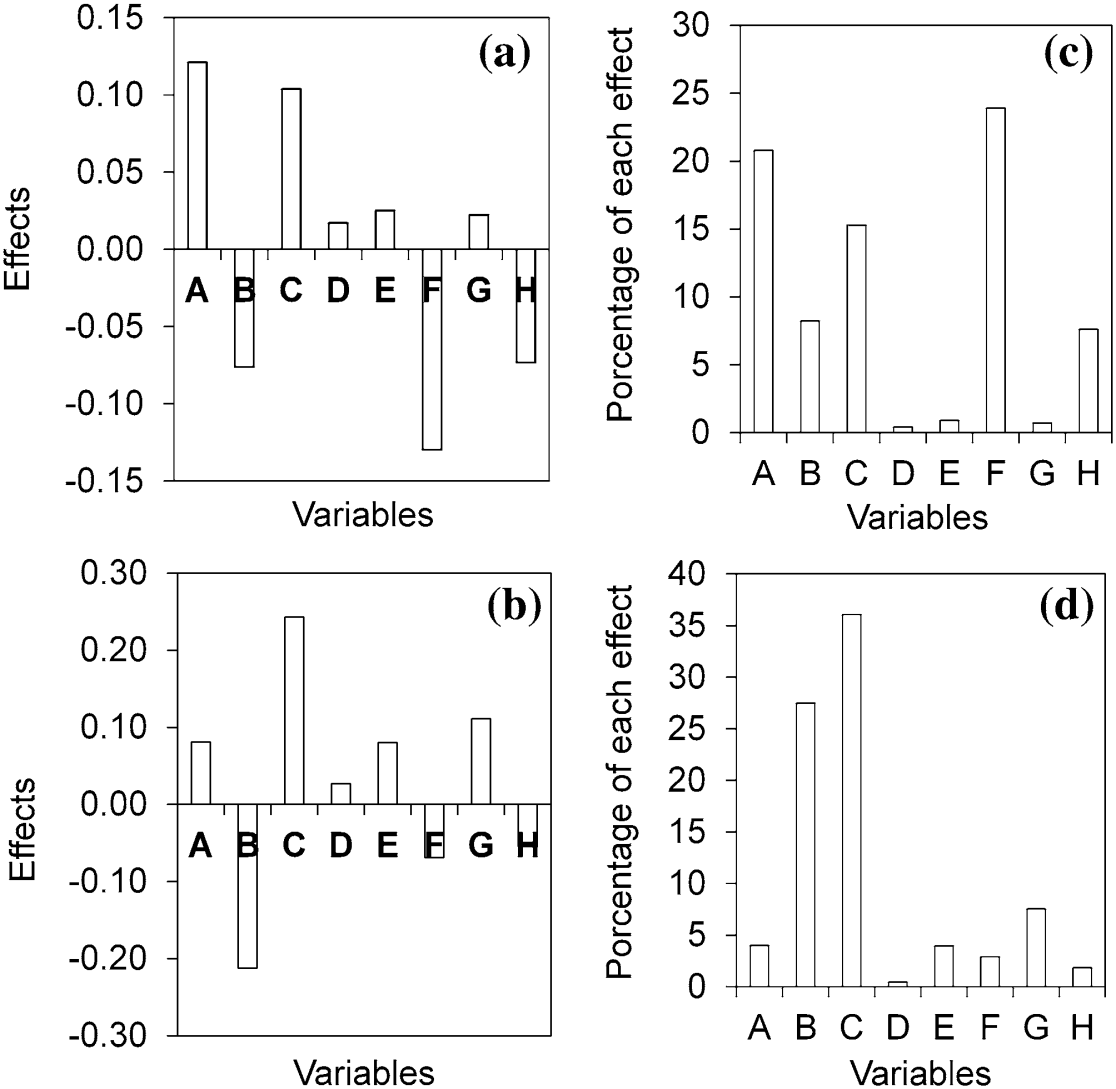
Graphic representation of the main differences in the investigated independent variables using gelatin (**a**) and lignin (**b**) as encapsulation materials. Percentage of each difference when using gelatin (**c**) and lignin (**d**) as the encapsulation materials. Evaluated variables: A: inlet air temperature; B: feed flow rate; C: drying air-flow; D: air injection flow; E: wall material content; F: adjuvant/polymer ratio; G: essential oil/polymer ratio; H: surfactant.

Thus, to optimize the use and efficiency of the spray-dryer for each evaluated polymer, we planned a fractional experiment. Table 1 shows the 16 experiments proposed by the experimental design, the response variables corresponding to each experiment, and the global desirability values. The effect of each variable on the orange EO encapsulation can be verified in Fig. 1.

**Table 1:** Influence of the used biopolymer (gelatin and lignin) on power recovery, oil content, and encapsulation efficiency. Experiments 1 to 16 refer to the fractioned factorial design.

Experiment	Powder recovery (%)		Oil content^a^ (%)		Encapsulation efficiency^a^ (%)		Global desirability	
	Gelatin	Lignin	Gelatin	Lignin	Gelatin	Lignin	Gelatin	Lignin
1	21.59	31.16	62.36	19.06	37.20	49.55	0.49	0.34
2	41.63	44.52	32.28	24.88	61.24	50.07	0.54	0.58
3	17.58	16.88	20.39	11.50	10.40	5.69	0.19	0.06
4	13.67	25.60	65.56	11.13	44.97	18.16	0.42	0.12
5	10.98	52.11	64.80	30.96	34.10	46.77	0.38	0.78
6	41.13	53.29	26.94	13.91	33.66	13.44	0.50	0.40
7	43.23	79.85	10.94	8.85	21.51	13.43	0.43	0.50
8	18.54	59.02	90.41	15.62	46.69	25.10	0.63	0.49
9	32.30	43.30	9.47	13.28	15.40	11.49	0.29	0.31
10	21.18	33.96	80.54	27.65	49.48	96.72	0.60	0.56
11	15.09	32.66	71.20	12.58	48.38	18.64	0.47	0.21
12	25.68	34.96	13.13	12.55	9.37	12.44	0.24	0.23
13	42.78	62.34	13.61	13.98	16.03	24.16	0.44	0.48
14	44.25	72.70	53.47	23.62	97.00	28.93	0.70	0.78
15	7.56	50.48	86.40	13.92	19.67	44.68	0.48	0.38
16	49.98	54.13	9.84	20.14	23.37	41.79	0.50	0.55

The values from 1 to 16 refer to the experiments proposed by the factorial design; % in w w^−1^; ^a^ values referent to d-limonene.

Based on the experimental data, we found that, when using gelatin as the biopolymer (Fig. 1a,c), the variables feed flow rate (B), air injection flow (D), wall material content (E), EO/polymer ratio (G), and surfactant (H) showed negligible effects (less than 10%), Fig. 1c. Only the inlet air temperature (A), drying air-flow (C), and adjuvant/polymer ratio (F) showed significant effects on the drying process (Fig. 1c).

The positive effect of temperature (Fig. 1a) indicates better oil content and higher powder recovery with higher drying air temperatures. Similar results were described for the encapsulation of essential oils from orange^14^, oregano^19^, and rosemary^20^ when the authors used temperatures higher than 130 °C. The increase in temperature favored the drying process by reducing the surface tension and the viscosity of the material, assisting in the droplets formation^21,22^.

In this study, the drying air-flow also showed a positive effect on the oil content and powder recovery, in which an increase in the flow rate of heated air in the drying chamber favored the atomization process (Fig. 1a). In 2003, Bruschi et al.^23^ demonstrated that higher drying air flows allow the pulverized colloidal suspension to evaporate before contacting the surface of the drying chamber^24^, also improving the formation of products with lower moisture content.

The adjuvant/polymer ratio was the only independent variable that had a negative effect (Fig. 1a); that is, the addition of adjuvant in the formulation did not improve the atomization process. In general, the use of technological adjuvants ("auxiliary dryers"), such as colloidal silicon dioxide, maltodextrins, and cyclodextrins, influence the powder recovery of the drying process and the redispersion of the powdered products in the colloidal suspension^25–27^.

For the products obtained using lignin as the encapsulation material, we verified that the drying air-flow and the feed flow rate showed the greatest influences on the process, resulting in a better atomization efficiency (Fig. 1b,d). The combination of the lower sample transfer rate (0.15 L h^−1^) and the higher drying air flow rate (536 L h^−1^) resulted in better powder recovery and oil content in this atomization process (Table 1).

Comparing the results obtained for both biopolymers, we can observe that the highest oil content ranged between 30 and 90% (w w^−1^) (Table 1). The oil content of up to 41% was reported using gelatin as wall material in propolis encapsulation^23^. In the literature, we observed values ranging from 71 to 98% in the encapsulation of orange essential oil by spray-drying process using Arabic gum, or polymeric combinations, repectively^14,15^.

We could also verify that lignin promoted the best powder recovery (Table 1). We obtained powder recovery between 44 and 72% (w w^−1^) for gelatin and lignin, respectively. Theses powder recoveries were obtained in experiments with global disability values higher than 0.70 (Table 1). In your turn, Márquez-Gómez et al. (2018)^14^ described powder recoveries of 29, 36, and 73% by using maltodextrin, hydrolyzed protein, and rice starch, respectively, for encapsulation of orange essential oil. These data indicate that gelatin and lignin are a viable alternative for the encapsulation of orange EO.

Additionally, we evaluated the encapsulation efficiency (Table 1). Through these data, we observed EO recoveries ranging from 5 to 97% (w w^−1^) according to each experimental condition proposed by experimental design. Values higher than 90% confirm the retention capacity of orange essential oil in gelatin and lignin microparticles when they were applied as wall materials. Moreover, these data suggest that there was only a small loss of EO during emulsion production and atomization drying, indicating the efficiency of the proposed method.

In this way, for the next steps of this study, emulsions with both biopolymers were prepared without the addition of a surfactant and colloidal adjuvant. They were subjected to atomization drying with a flow transfer rate of 0.15 L h^−1^, drying air-flow rate of 536 L h^−1^, and inlet air temperature of 150 °C.

### Microparticle characterization

**Figure 2:**
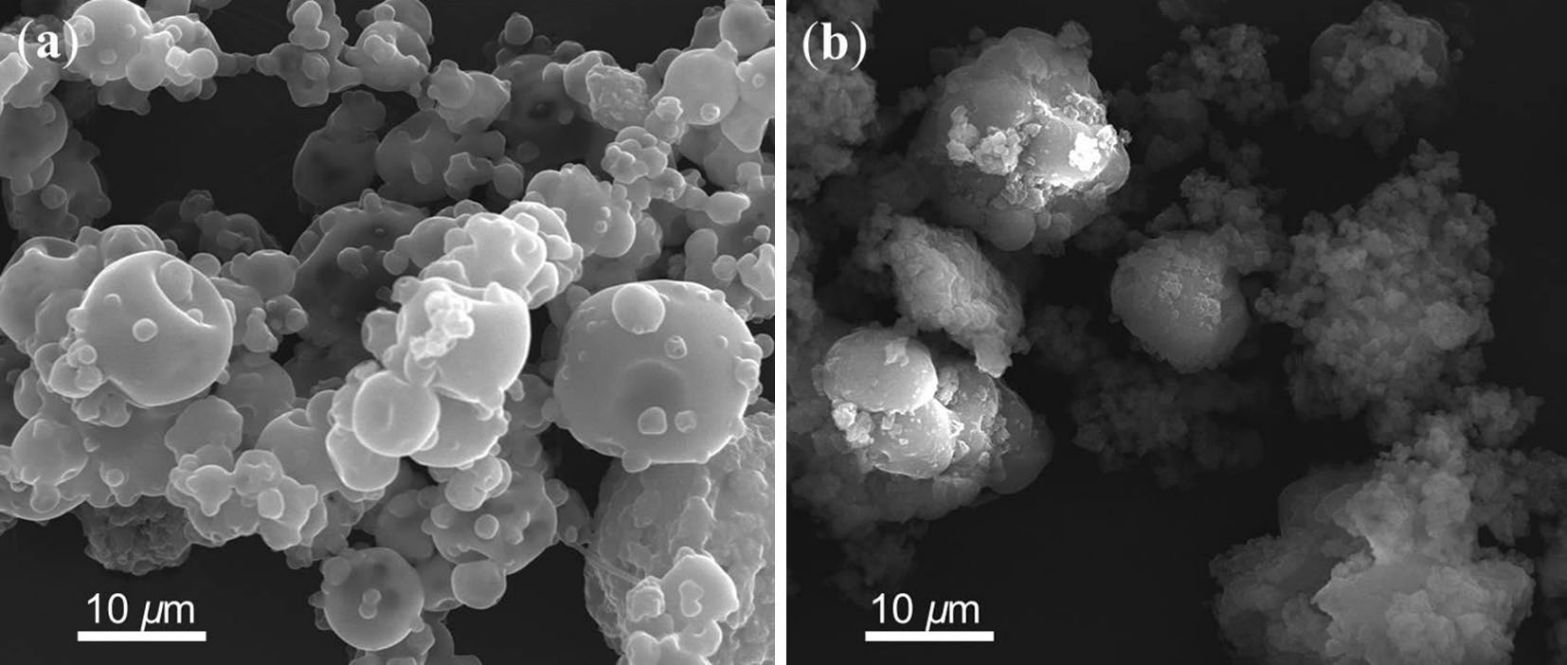
Microphotographs of the microparticles loaded with orange essential oil produced by spray-drying using gelatin (**A**) and lignin (B**)** as the biopolymers (with × 5,000 of magnification).

The microparticles obtained by using gelatin presented a microspherical structure with a smooth and continuous surface, which is fundamental to prevent the penetration of gases and protect the nucleus (Fig. 2a)^11^. These morphological characteristics relate to the ability of gelatin to form a continuous film around the droplets of essential oil before the drying process^28,29^. The gelatin microparticles also did not present cracks, which could cause loss of the volatile fractions of the microencapsulated compounds^11^ because of their permeability^20^.

When we used lignin as the biopolymer, the microparticles presented an irregular and rough structure characteristic of the biopolymer used (Fig. 2b)^11^. Although lignin has emulsion-stabilizing properties^12^, it showed irregular morphological characteristics that can be a challenge for its application in microencapsulation processes due to the wide diversity of functional groups, which may vary according to its botanical and industrial origins^12^.

Although both polymers showed some agglomeration of microparticles, it was still possible to identify individual microparticles, indicating that the agglomerates were formed after the complete drying of each microparticle. Similar results were obtained in the encapsulation of chia and oregano essential oils^19,30^.

The microparticles also presented a heterogeneous size distribution showing a peak of frequency under 4.0 μm. Particles containing gelatin as wall material showed a larger average particle size (3.69 μm) compared to particles containing lignin (2.71 μm) (Supplementary Material Fig. S1). Moreover, more than 50% of the gelatin particles presented sizes 4 and 10 μm. The largest average particle size may be related to the higher solubility and emulsification capacity of this polymeric material. Thus, lignin, which has a lower solubility in an aqueous medium than gelatin, produces smaller particles with matrix characteristics^10^.

According to the data that we obtained through our experimental design and the morphological analyses, we observed that the microparticles obtained by using gelatin and lignin as biopolymers were able to load the orange essential oil. Although the best results of oil content were obtained with gelatin, we would like to highlight the lignin formulations, which also showed good results. To the best of our knowledge, this is the first work describing the microencapsulation of essential oils by using lignins, a waste product from the pulp and paper industries. Moreover, lignin can act as a natural antioxidant^31^ and has the potential to incorporate antimicrobial activity and photostability in the final microencapsulated product. Additionally, after the method was optimized, the powder recovery of the final product was almost three times higher with lignin than that obtained with gelatin.

### Quantitative analyses of the microencapsulated essential oil by gas chromatography

In addition to the encapsulation stage, the quantification of the encapsulated essential oil is an important step to evaluate the quality of the final product. The different intrinsic properties between the essential oils (low molecular weight and have high vapor pressure^1^) and the biopolymers (macromolecules with properties that are the opposite of essential oils^32,33^) present a challenge in developing a robust quantitative method that is compatible with both materials. Among the different techniques for sample preparation and analyses, the quantification by gas chromatography with headspace extraction is a good alternative, due to reduce the possibility of evaporation losses during sample preparation steps^34^, ensuring the transference only of volatile organic compounds.

Thus, to evaluate the performance of the developed quantitative method by headspace gas chromatography (headspace GC-FID) and to ensure its reliability, we carried out several analyses that investigated parameters such as selectivity, matrix effects, limits of detection and quantification, linearity, precision, and accuracy.

### Selectivity

**Figure 3:**
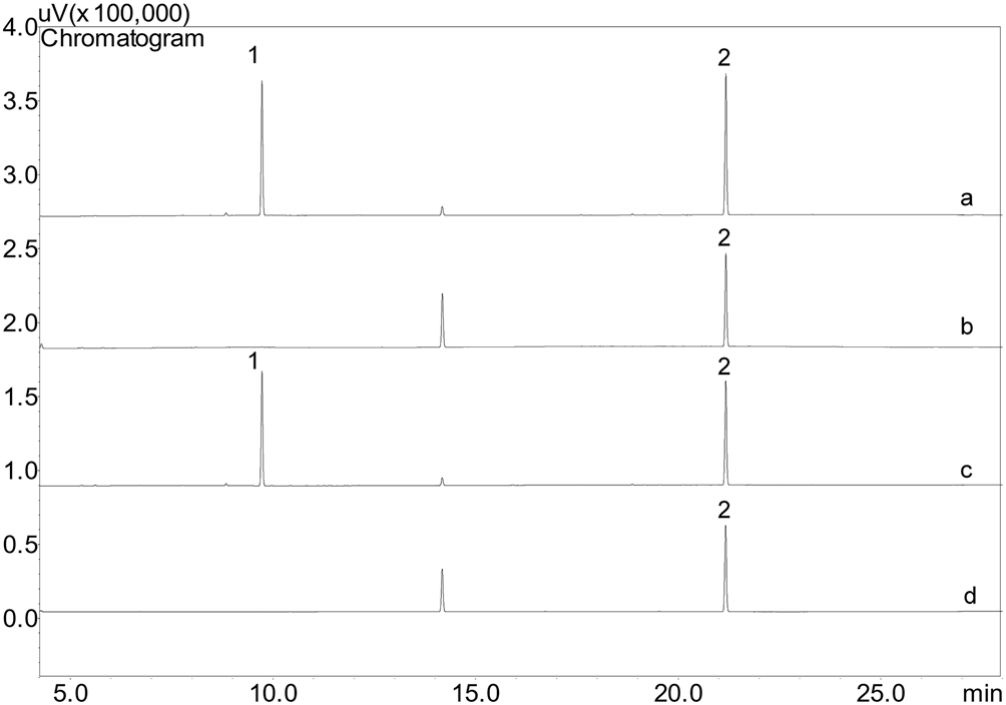
Chromatograms of the microparticles of gelatin (**A**) and lignin (**C**) loaded with d-limonene (1.0 µg mL^−1^), and microparticle controls of gelatin (**B**) and lignin (**D**) without d-limonene. Identification of the peaks: (1) d-limonene and (2) menthol (IS).

The chromatograms of the control samples (orange essential oil-free microparticles) and the gelatin and lignin microparticles containing orange essential oil showed no interference peaks from the matrices or the internal standard that affected the retention time of the analyte (Fig. 3). Therefore, the developed method was shown to be selective for the analysis of d-limonene and could distinguish the response of the analyte of interest (d-limonene) from the other responses produced by the components from the matrices (i.e., the biopolymers)^35^.

### Evaluation of matrix effects in the d-limonene analysis by headspace gas chromatography

**Figure 4:**
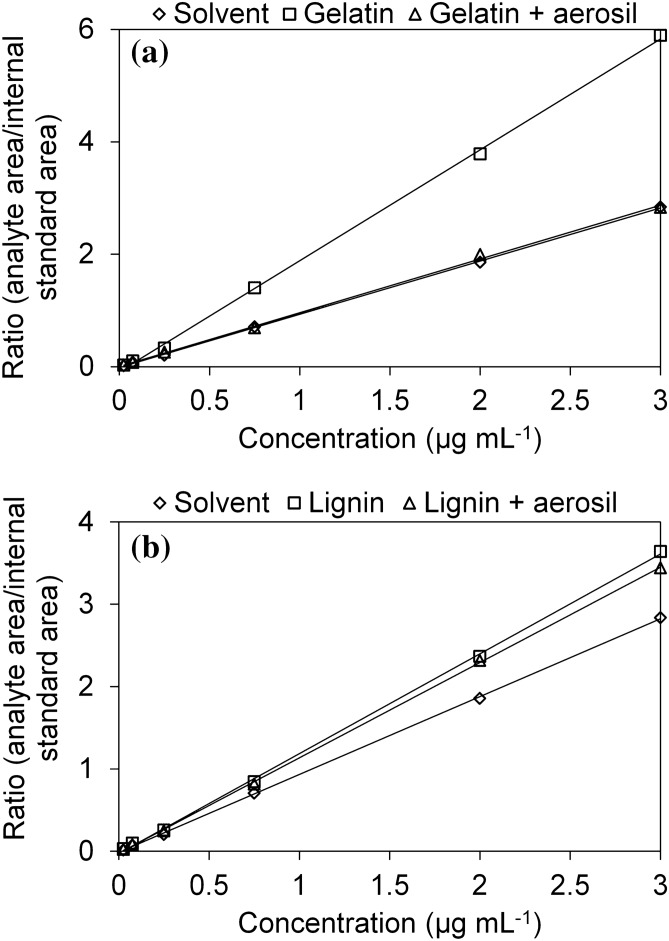
Analytical curves for d-limonene prepared in solvent (acetone) and matrices. (**a**) Gelatin and gelatin:Aerosil. (**b**) Lignin and lignin:Aerosil.

In this study, we investigated the matrix effects that are directly related to the headspace extraction step. To evaluate the matrix effects on the headspace extraction of d-limonene, we carried out a matrix-matched calibration. The influence of co-extractives on the d-limonene headspace extraction could be observed through the slope differences among the analytical calibration curves, which were obtained for lignin, gelatin, and their combination with a colloidal adjuvant, as illustrated in Fig. 4.

The ratios between the angular coefficients obtained in the calibration curves in the matrix and solvent (Supplementary Table S2) were higher than 1.00, indicating a positive matrix effect. The matrix effects could also be observed by variations in the quantification of the analyte in the powdered products according to the prepared calibration curve in the matrix or pure solvent (Supplementary Fig. S2). When we did not include the matrix effects, the essential oil recoveries were greater than 150%, for example, in the gelatin formulations. Clearly, this is caused by matrix effects. Thus, when we included the interference of the matrix components, there was variation in the chromatographic signal for d-limonene, with variations as high as 96% and 89% for the gelatin and lignin formulations, respectively.

These data indicate that the presence of the matrix components promotes greater extraction in the headspace with an increased chromatographic signal for d-limonene. This finding may be explained by partial solvent vaporization during headspace extraction. In the presence of the matrix components, the solvent may interact with and/or dissolve other molecules present on the matrices, decreasing its concentration in the vapor phase, and assisting in the release and extraction of d-limonene (thereby increasing the coefficient of volatilization of d-limonene).

Therefore, the quantitative analyses of microparticles loaded with d-limonene were carried out using analytical calibration curves prepared in the presence of the matrices to minimize their effects and to guarantee the accuracy of the analytical method.

### Linearity and limits of detection and quantification of the GC-FID analytical method

The linearity of the proposed method was evaluated according to the coefficient of determination (*r*^2^) from the calibration curves prepared in the presence of the biopolymer matrices of gelatin and lignin, and in the acetone solvent (Fig. 4). The calibration curves were linear, and covered a range from 0.025 to 3.00 µg mL^−1^ with coefficients of determination (*r*^2^) greater than 0.99 regardless of the matrix effects, indicating a good correlation between the area of the d-limonene peak and its concentration^36^.

In this study, we also found that when using gelatin as the matrix, the calibration curve presented a higher analytical sensitivity to detect variations in the concentration of d-limonene. The analysis of variance (ANOVA), the graphs of the residues for the calibration curve samples in relation to the adjusted regression and the Cochran’s C test for homogeneity (95% confidence level) are presented in the supplementary material (Supplementary Table S3 and S4 and Supplementary Fig. S3).

The LOD and LOQ values obtained (Table 2) verified that the analytical calibration curve prepared by using extracts from the gelatin matrix showed values of LOD and LOQ that were approximately twice those of the values obtained in the solvent. These data confirmed the stronger matrix effect on the analytical sensitivity when gelatin was used as biopolymer^37^. The sensitivity and linearity of the method were also demonstrated by the low values of the obtained LOD, which were significantly lower than the minimum necessary for the quality control of the microparticles loaded with d-limonene.

**Table 2:** Limit of detection (LOD) and quantification (LOQ), the linearity of the method, and power recovery percentages of d-limonene after extraction of gelatin and lignin samples spiked with three different concentrations.

Matrix	Equation	*r*^2^	LOD	LOQ	%R ± RSD Concentration (µg mL^−1^)		
			(mg g^−1^)		0.30^a^	1.2^a^	2.4^b^
Acetone	y = 0.941x − 0.009	0.999	0.051	0.153	–	–	–
Gelatin	y = 1.972x − 0.086	0.998	0.024	0.073	103 ± 3.3	105 ± 5.5	111 ± 7.1
Lignin	y = 1.212x − 0.025	0.995	0.039	0.119	99.1 ± 3.1	109 ± 6.0	103 ± 13

^a^Three repetitions; ^b^seven repetitions. *r*^2^ coefficient of determination, *LOD* limit of detection, *LOQ* limit of quantification, *R* recovery, *RSD* relative standard deviation.

### Precision and accuracy

The ability of the developed analytical method to present the concentration–response relationship (linearity) and reproducibility was evaluated by systematically repeating analyses of known samples.

The precision was expressed by the Relative Standard Deviation (RSD) obtained for seven identical headspace extractions of fortified samples with 2.4 µg mL^−1^ of d-limonene, using lignin and gelatin as the biopolymers. Table 2 describes the RSD values, which were lower than 15% for both biopolymers (values of 7.1 and 13% for gelatin and lignin, respectively). These results demonstrate the reproducibility of the method. RSD values that are close to or smaller than the recommended (RSD < 20%) indicate the low influence of random errors, which is considered to be acceptable in ensuring the precision of the method^38,39^.

The accuracy of the analytical method was evaluated by comparing the nominal and experimental values that were obtained by the quantification d-limonene in the samples within the linear range of the calibration curves, at three different concentration levels (low, medium, and high), which were prepared independently in triplicate. Table 2 shows the accuracy and RSD values of the three concentration levels. For all matrices, the method showed good performance, with accuracy between 99 and 111%, which indicated an agreement among the experimental and theoretical values and a low influence of systematic errors.

## Conclusion

In this work, formulations to encapsulate orange essential oil were successfully obtained using spray-drying process. Fractional factorial design indicated that the main variables that should be controlled to obtain the best powder recovery and oil content were the sample flow transfer rate, drying air-flow, and temperature.

Working under the optimized conditions, concluded that it is possible to prepare microparticles by spray-drying using gelatin and lignin as biopolymers. It was possible to prepare microparticles showing microspherical morphology, with the average particle size of less than 4 µm (gelatin) and 3 µm (lignin), with an oil content of 90% (w w^−1^) for gelatin and powder recovery of 72% (w w^−1^) for lignin as wall material.

The quantification method development and validation were compatible with the intrinsic features of the essential oil and the biopolymers. The matrix effects were addressed by using matrix-matched calibration curves. In doing so, the analytical method was validated, showing linearity, selectivity, precision, and accuracy for the quantitative analysis of d-limonene. Additionally, the analytical method presented advantages such as its operational simplicity. The analytical method also assisted in the optimization of the experimental variables for the production of microparticles loaded with essential oil by providing quantitative data.

## Experimental section

### Chemicals

Stock standard solutions of d-limonene (99% w w^−1^), purchased from Sigma-Aldrich (St. Louis, United States of America), and menthol as an internal standard (Arora Chemicals, Sao Marcos, Brazil) were prepared in acetone at 5.0 and 8.0 mg mL^−1^, respectively. Both solutions were stored at − 4 °C.

The solvents used were acetone of HPLC grade (Panreac, Barcelona, Spain) and ultrapure water (Milli-Q, Millipore). Gelatin type B (Synth, Sao Paulo, Brazil) and lignin (provided by Suzano S/A, Sao Paulo, Brazil) were used as the encapsulating materials. The surfactant sorbitan monostearate (Span 60, Sigma-Aldrich, St. Louis, United States of America) and the adjuvant colloidal silicon dioxide (Aerosil 200, Evonik, Essen, Germany) were also used in the formulations.

### Sample

The orange essential oil, produced by cold pressing the orange peel, was obtained from the region of Santa Cruz do Rio Pardo—SP (Agroterenas S/A Citrus, Brazil). This EO was used as the volatile core material. The specifications of the EO were as follows: density at 25 °C: 0.842 g mL^−1^, the refractive index at 20 °C: 1.473, and aldehydes: 1.4%.

To evaluate the volatile composition, the volatile compounds were analyzed by a gas chromatograph hyphenated with a sequential mass spectrometer (Shimadzu Corporation, Japan TQ8040). In these analyses, we used a capillary column (Rtx-5MS, Restek) with a stationary phase consisting of 5% diphenyl and 95% dimethylpolysiloxane (30 m × 0.25 mm d.i. × 0.25 µm film thickness) and Helium 5.0 as carrier gas (1 mL min^−1^). The chromatographic conditions were as follows: the injector temperature was 220 °C; the initial oven temperature for the column was 60 °C (1 min), then it was heated at a rate of 3 °C min^−1^ to 230 °C and maintained at this temperature for 1 min. The interface transfer temperature was 240 °C; the injection mode was split (100:1); the injected sample volume was 1.0 µL. The electron impact mass spectra were recorded at 70 eV ionization energy, with the acquisition quadrupole mass ranging from 40 to 700 a.m.u., in scanning mode, with 0.3 scans per second. The volatile organic compounds were identified by comparison with a library of spectral data (NIST 17.0), the literature data described by Adams (2009)^40^, and the retention index as proposed by Van den Dool e Kratz^40^.

### Orange essential oil microencapsulation

Multivariate optimization was applied to determine the optimal condition for drying and encapsulating orange EO by atomization (Spray-drying). For this purpose, we employed fractional factorial design with eight independent variables (2^8–4^), totaling 16 experiments, with the experiments randomly conducted to minimize the effects of unexpected variability in the observed responses due to systematic errors. The following variables were selected to optimize the spray-dryer performance, with high and low increments: inlet air temperature, feed flow rate, drying air-flow, air injection flow (Aspirator), wall material content, adjuvant/polymer ratio, essential oil/polymer ratio, and surfactant. The levels evaluated are described in Table 3. Those parameters were selected from previous studies in our research group, and by seeks in literature^14,19,41,42^. We evaluated the use of gelatin and lignin as the biopolymers.

**Table 3:** Coded levels of the independent variables, and matrix representation of the fractional factorial design (2^8–4^).

	Variables	Levels	
		− 1	+ 1
A	Inlet air temperature (°C)	110	150
B	Feed flow rate (L h^−1^)	0.15	0.45
C	Drying air flow (L h^−1^)	301	536
D	Air injection flow (m^3^ h^−1^)	8	35
E	Wall material content (% w v^−1^)	5	10
F	Adjuvant/polymer ratio (w w^−1^),	1:0	1:1
G	Essential oil/polymer ratio (w w^−1^)	1:1.78	1:3.56
H	Surfactant (mg)	0	200

Experiment	Coded levels							
	A	B	C	D	E	F	G	H
1	− 1	− 1	− 1	− 1	− 1	− 1	− 1	− 1
2	+ 1	− 1	− 1	− 1	+ 1	+ 1	+ 1	− 1
3	− 1	+ 1	− 1	− 1	+ 1	+ 1	− 1	+ 1
4	+ 1	+ 1	− 1	− 1	− 1	− 1	+ 1	+ 1
5	− 1	− 1	+ 1	− 1	+ 1	− 1	+ 1	+ 1
6	+ 1	− 1	+ 1	− 1	− 1	+ 1	− 1	+ 1
7	− 1	+ 1	+ 1	− 1	− 1	+ 1	+ 1	− 1
8	+ 1	+ 1	+ 1	− 1	+ 1	− 1	− 1	− 1
9	− 1	− 1	− 1	+ 1	− 1	+ 1	+ 1	+ 1
10	+ 1	− 1	− 1	+ 1	+ 1	− 1	− 1	+ 1
11	− 1	+ 1	− 1	+ 1	+ 1	− 1	+ 1	− 1
12	+ 1	+ 1	− 1	+ 1	− 1	+ 1	− 1	− 1
13	− 1	− 1	+ 1	+ 1	+ 1	+ 1	− 1	− 1
14	+ 1	− 1	+ 1	+ 1	− 1	− 1	+ 1	− 1
15	− 1	+ 1	+ 1	+ 1	− 1	− 1	− 1	+ 1
16	+ 1	+ 1	+ 1	+ 1	+ 1	+ 1	+ 1	+ 1

(− 1) lower level; (+ 1) higher level.

In this way, colloidal suspensions were progressively produced by the addition of known quantities of biopolymer (mg), surfactant (mg), colloidal adjuvant (mg), and essential oil (mg) into 30 mL of ultrapure water in a 125 mL Erlenmeyer flask (Table 1). The system was maintained under magnetic stirring at 20,000 rpm for 60 s using a disperser (Ultra-Turrax IKA T10 basic, Wilmington, United States America) at 20 °C (± 1 °C).

The colloidal suspensions were immediately transferred to a spray-dryer (Mini *Spray Dryer* BÜCHI, B 290, Flawil, Switzerland) equipped with a drying chamber (500 mm × 200 mm) and a nozzle atomizer (0.7 mm). The resulting dried products were collected, kept glass desiccator during 48 h, and then stored under refrigeration (8 °C).

The powder recovery (% w w^−1^) in the encapsulation process was calculated according to the Eq. (1):

1 $${\text{Powder }}\;{\text{Recovery}}\;{ }(\% ) = \frac{{{\text{W}}_{{\text{S }}} }}{{{\text{W}}_{{{\text{S}}0}} }} \times 100$$

where W_S_ is the mass of dried material collected after drying in the spray-dryer and W_S0_ is the initial quantity of solids in the sprayed emulsion volume.

### Oil content and encapsulation efficiency

The total orange essential oil quantity in the microencapsulated products that were obtained by spray-drying was evaluated by quantifying the d-limonene content present in the internal phase (the encapsulated nucleus) and adsorbed onto the surface of the particles after solubilizing 25.0 mg of the dried material in 1,000 µL of acetone for 30 min. To 800 μL of the resulting mixture, 100 μL of menthol (1,000 µg mL^−1^, IS) was added. The samples were extracted by static headspace (SH). SH analysis was performed using a PALSyr HS 2.5 mL for combi-PAL. To do so, 10 µL of the sample was transferred into a headspace vial (10 mL) and homogenized for 15 min at 75 °C and 500 rpm. Subsequently, 1,000 µL of the vapor phase was injected into GC.

Quantitative GC analyses were carried out using a Shimadzu (GC 2010 Plus) apparatus coupled with a Flame Ionization Detector (FID). Analyses were carried out using a ZB-Wax capillary column (30 m × 0.25 mm i.d.; 0.25 µm film thickness) coated with polyethylene glycol (Phenomenex). The programmed oven temperature was 40 °C for 1 min, raised at 5 °C min^−1^ to 170 °C and held for 1 min; the injector temperature was 170 °C; the carrier gas was Helium 5.0 at a constant flow rate of 1 mL min^−1^; the injection mode was split (15:1). Synthetic air (300 mL min^−1^), hydrogen (40 mL min^−1^) and nitrogen (30 mL min^−1^) were used for FID.

To evaluate the content of the essential oil in the powder products, at the end drying process (oil content %, w w^−1^), we determined the concentration of d-limonene using the data from an analytical curve obtained by the analyses of standard solutions in concentrations ranging from 0.025 to 3.00 µg mL^−1^. The encapsulation efficiency (% w w^−1^) was obtained comparing the oil content, in the powder products to the used quantity of OE in the preparation of each emulsion. The oil content (% w w^−1^) and the encapsulation efficiency (% w w^−1^) were calculated using the Eqs. (2) and (3), respectively:

2 $${\text{Oil }}\;{\text{Content}} \;(\% ) = \frac{{{\text{W}}_{{\text{E }}} }}{{{\text{W}}_{{\text{S}}} }} \times 100$$

3 $${\text{Encapsulation }}\;{\text{Efficiency}}\; (\% ) = \frac{{{\text{W}}_{{\text{E }}} }}{{{\text{W}}_{{{\text{E}}0}} }} \times 100$$

where W_E_ is the mass of EO obtained after drying and W_E0_ is the mass of the EO used during the preparation of emulsions.

### Global desirability function for the development of essential oil-loaded formulations

To relate the levels of the dependent variables (powder recovery and oil content) and find the best conditions that produced the most desirable responses, we used the global desirability function^25^. This function allows for the normalization of the values obtained from the individual desirability values (d_i_)^25,43,44^ and obtains the global desirability value (D) for each experiment by calculating the geometric average of the d_i_ values^44^. The desirability values are on a scale from 0 to 1, where values closer to 1 are considered desirable^43^. The desirability values were also used to calculate the main effects and interaction of the variables for the microencapsulation of orange EO.

### Microparticle morphology evaluation

The surface morphology of the microparticles was visualized using scanning electronic microscopy (SEM). The powdered samples were fixed to a double-sided adhesive carbon tape mounted on SEM stubs with a diameter of 12 mm (Koch, Sao Paulo, Brazil), and then coated with a thin layer of gold/palladium in a vacuum. The images were magnified to 200 to 50,000 × under an FEI Inspect S50 microscope operating at 25 kV^41^. The particle size was measured by analyzing the images with ImageJ software^45^. The particle size distribution was determined by measuring the diameter of approximately 300 particles. The resulting values were plotted on a histogram and adjusted with a Gaussian function.

### Analytical method validation

The selectivity, linearity, limits of detection (LOD) and quantification (LOQ), accuracy, and precision are parameters that were used to evaluate the analytical method for the quantification of the orange essential oil^38,39,46^.

The linearity was verified by preparing standard solutions of increasing concentrations of the analyte d-limonene in solution (0.025, 0.075, 0.250, 0.750, 2.00 and 3.00 µg mL^−1^) and also using a solid matrix (gelatin, gelatin and aerosil, lignin, and lignin and aerosil) as the dispersal medium, and an internal standard (1.0 µg mL^−1^). Thus, for each condition, 10 µL of the standard solution was transferred into a headspace vial (10 mL) and homogenized for 15 min at 75 °C and 500 rpm. Subsequently, 1,000 µL of the vapor phase was injected into the GC.

After the analysis of these solutions, a graph was constructed relating the ratio between the area of the analyte and the internal standard to their respective concentration used in the calibration. The linearity was evaluated by the determination coefficient (*r*^2^) calculated by linear regression and verified by analysis of variance regarding regression, accuracy, and analytical recovery. The evolution of the matrix co-extractives in the chromatographic signal was evaluated by the relationship between the area of the analyte in the pure solvent (acetone) and the area obtained using the standard prepared in aqueous extracts of the matrix (matrix-matching), where the matrix effect (%) = (Ā_matrix _ – Ā_solvent_/Ā_solvent_) × 100). Extracts of the matrix (gelatin, gelatin and Aerosil, lignin, and lignin and Aerosil) were also used as a dispersal medium for the preparation of the curves to determine the matrix effects.

The selectivity was observed by evaluating the chromatograms of the matrix that were obtained after the extraction of the components in the analyte-free matrix compared to the chromatograms of the extracts of the microparticles that were prepared and analyzed according to the optimized procedure.

The LOD and LOQ for d-limonene were determined for each matrix by successively diluting the stock solution of d-limonene and by the parameters of the analytical curve, using the following equations: LOD = (3.3*δ*)/*S* and LOQ = (10*δ*)/*S*, where *δ* is the standard deviation of the calculated controls by analyzing the noise of three samples and *S* is the slope of the calibration curve.

The method precision was determined by calculating the relative standard deviation and the coefficient of variation of seven formulations in identical extractions of the orange EO at a concentration of 2.4 µg mL^−1^. The extraction recoveries (%R) were determined in samples of microparticles containing d-limonene at different levels: 0.30, 1.2, and 2.4 µg mL^−1^. The accuracy was evaluated by the recovery of the analyte.

### Statistical analysis

Analysis of variance (ANOVA), multiple comparisons of means by the Tukey test (5% probability), and the Cochran test (5% probability) were performed using SPSS software (Statistical Package for Social Science for Windows), version 17.0.0 (2008).

## Supplementary information

Supplementary Information
