# Supplementary material for: Evaluation of the microencapsulation of orange essential oil in biopolymers by using a spray-drying process
Source: Sci Rep. 2020 Jul 16;10:11799. doi: 10.1038/s41598-020-68823-4 (PMC7367259; doi:10.1038/s41598-020-68823-4)
Supplement: Supplementary file 1 — Supplementary Information [file 41598_2020_68823_MOESM1_ESM.docx]

**Evaluation of the Microencapsulation of Orange Essential Oil in Biopolymers by Using a Spray-Drying Process**

Maria Clara Santana Aguiar^a^, Maria Fátima das Graças Fernandes da Silva^a^, João Batista Fernandes^a^, Moacir Rossi Forim^a,^*

^a^*Department of Chemistry, Federal University of São Carlos, Rod. Washington Luiz, Km 235, Postal Code 13565-905, São Carlos - SP, Brazil*

*Corresponding author

Moacir Rossi Forim, Tel: +55 16 3351.8061; Fax: +55 16 3351.8350

e-mail: *mrforim@ufscar.br*

**Table S1.** Relative areas of the compounds identified in orange EO.

| Compound | R_t_  (min.) | RI | RI_Ref_ | RA (%) | [M] | Main fragments *m/z* (%) |
| --- | --- | --- | --- | --- | --- | --- |
| Heptanal | 5.2 | 903 | 901 | 0.01 | 114 | 55(64.64); 57(58.14); 70(96.54); |
| *α*-pinene | 6.1 | 934 | 932 | 3.99 | 136 | 77(26.53); 79(24.16); 91(51.15); 93(100); 105 (10.71); 136(6.54) |
| Camphene | 6.5 | 950 | 946 | 0.02 | 136 | 67(27.00); 77(20.72); 79(41.19); 91(38.66); 93(100); 107(30.80); 121(66.67) |
| Heptanol | 7.1 | 968 | 959 | 0.01 | 116 | 41(72.26); 42(53.24); 43(67.26); 55(78.60); 56(83.15); 57(22.52); 70(100) |
| Sabinene | 7.3 | 974 | 969 | 1.80 | 136 | 41(20.88); 77(37.74); 79(28.41); 80(12.85); 91(52.44); 93(100); 136(12.97) |
| *β*-pinene | 7.4 | 979 | 974 | 0.12 | 136 | 41(36.45);69(29.07); 77(20.84); 79(24.67); 80(13.57); 91(32.60); 93(100); 136(5.58) |
| *β*-myrcene | 7.8 | 992 | 988 | 11.1 | 136 | 41(92.25); 67(13.48); 69(67.75); 77(15.53); 79(16.89); 91(31.35); 93(100) |
| Octanal | 8.2 | 904 | 998 | 1.55 | 128 | 41(87.87); 43(100); 44(75.71); 56(74.06); 69(39.49); 84(50.77) |
| *α*-thujene | 8.2 | 907 | 924 | 0.02 | 136 | 41(11.84); 77(33.69); 91(65.49); 93(100); 136(24.25) |
| 3-carene | 8.5 | 1013 | 1008 | 0.57 | 136 | 77(30.89); 79(32.29); 91(57.58); 93(100); 105(14.05); 121(19.31); 136(12.38) |
| *d*-limonene | 9.2 | 1031 | 1024 | 77.5 | 136 | 67(89.91); 68(100); 79(40.97); 93(88.01); 94(46.36); 107(27.69); 121(27.38); 136(19.23) |
| *E-β*-ocimene | 9.8 | 1048 | 1044 | 0.03 | 136 | 41(28.51); 79(51.57); 91(60.97); 93(100); 105(16.79); 121(13.63) |
| *γ*-terpinene | 10.2 | 1060 | 1054 | 0.01 | 136 | 44(27.97); 77(28.25); 91(73.63); 93(100); 107(17.70); 121(22.60); 136(37.95) |
| n.i. | 10.6 | - | - | 0.22 | - | 41(81.64); 43(64.90); 55(79.33); 56(100); 69(65.50); 70(55.93); 83(34.82); 84(40.21); |
| 2-carene | 11.4 | 1091 | 1001 | 0.04 | 136 | 41(23.05); 79(36.87); 91(57.42); 93(100); 105(22.48); 121(69.89); 136(61.55) |
| Linalool | 11.8 | 1101 | 1095 | 1.44 | 154 | 41(73.51); 43(72.28); 55(63.32); 71(100); 80(38.88); 93(92.68); 121(23.06); |
| Nonanal | 12.0 | 1106 | 1100 | 0.09 | 142 | 41(78.30); 43(64.03); 57(100); 70(40.84); 82(33.74); 98(31.48); |
| n.i. | 13.2 | - | - | 0.37 | - | 43(100); 67(82.21); 109(43.37); 137(33.52) |
| n.i. | 13.4 | - | - | 0.15 | - | 43(100); 67(66.99); 79(51.25); 94(61.87); 108 (41.81) |
| Citronellal | 14.0 | 1154 | 1153 | 0.05 | 154 | 41(100); 55(44.43); 69(76.01); 95(50.19); 121(27.93) |
| Menthol^*^ | 14.9 | 1174 | 1167 | 0.25 | 156 | 55(44.03); 71(90.33); 81(100); 95(82.36); 109(12.83); 123(31.22); 138(12.56) |
| *α*-terpineol | 15.6 | 1193 | 1186 | 0.06 | 154 | 59(100); 93(84.05); 121(58.83); 136(49.53) |
| Decanal | 16.3 | 1207 | 1201 | 0.27 | 156 | 43(100); 57(95.40); 82(54.26); 112(22.89) |
| Neral | 17.8 | 1243 | 1235 | 0.04 | 152 | 41(100); 69(71.66); 109(22.76) |
| *d*-carvone | 18.0 | 1247 | 1239 | 0.02 | 150 | 54(48.69); 82(100); 93(43.58); 108 (41.02) |
| Geranial | 19.1 | 1273 | 1264 | 0.06 | 152 | 41(100); 69(94.40); 109(10.77); 137(10.94) |
| Perilal | 19.3 | 1277 | 1269 | 0.02 | 150 | 41(43.20); 67(100); 79(87.03) 107(62.61); 135(35.64) |

R_t_: Retention time; n.i: Compound unidentified; RI: Retention index; RI_Ref_ : Retention index presented by ^25^; RA: Relative Area; [M]: Molecular mass; ^*^: added as internal standard.

**Figure S1.** Distribution of particle size prepared in (a) gelatin and (b) lignin.

**Table S2.** Equations and relationship between the linear and angular coefficients of the curves obtained in the matrices and solvent (acetone).

| Matrix | Equation | ACm/ACs | LCm/LCs |
| --- | --- | --- | --- |
| Solvent | y = 0.941x - 0.009 |  |  |
| Gelatin | y = 1.972x - 0.086 | 2.10 | 9.77 |
| Gelatin + aerosil | y = 0.985x - 0.007 | 1.05 | 0.85 |
| Lignin | y = 1.212x - 0.025 | 1.29 | 2.90 |
| Lignin + aerosil | y = 1.148x - 0.026 | 1.22 | 3.00 |

ACm = angular coefficient of the matrix; ACs = angular coefficient of the solvent; LCm = linear coefficient of the matrix; LCs = linear coefficient of the solvent;

**Figure S2**. Chromatographic response variation after Headspace GC-FID analysis for *d*-limonene. The values from 1 to 16 refer to the extracts obtained from the experiments proposed by the factorial design

**Figure S3**. Residual plots for *d*-limonene curves prepared in solvent (a), gelatin:aerosol (b), gelatin (c), lignin:aerosol (d), and lignin (e).

**Table S3**. Variance analysis for linear model adjustment (95% confidence level)

| Curve | Source of variation | Sum of squares | Degrees of freedom | Mean squares | F | F_tab_ |
| --- | --- | --- | --- | --- | --- | --- |
| Acetone | Regression | 27.77 | 1 | 27.77 | 10934.42 | 4.49 |
|  | Residual | 0.04 | 16 | 0.00 |  |  |
|  | Total | 27.81 | 17 |  |  |  |
|  | Pure error | 0.03 | 12 | 0.02 | 0.08 | 3.26 |
|  | Lack of fit | 0.01 | 4 | 0.00 |  |  |
| Gelatin: aerosil | Regression | 99.82 | 1 | 99.82 | 6624.27 | 4.49 |
|  | Residual | 0.24 | 16 | 0.02 |  |  |
|  | Total | 100.06 | 17 |  |  |  |
|  | Pure error | 0.16 | 12 | 0.01 | 1.61 | 3.26 |
|  | Lack of fit | 0.08 | 4 | 0.02 |  |  |
| Gelatin | Regression | 86.71 | 1 | 86.71 | 7937.79 | 4.49 |
|  | Residual | 0.17 | 16 | 0.01 |  |  |
|  | Total | 86.89 | 17 |  |  |  |
|  | Pure error | 0.11 | 12 | 0.01 | 1.61 | 3.26 |
|  | Lack of fit | 0.06 | 4 | 0.02 |  |  |
| Lignin | Regression | 32.73 | 1 | 32.73 | 3145.24 | 4.49 |
|  | Residual | 0.17 | 16 | 0.01 |  |  |
|  | Total | 32.89 | 17 |  |  |  |
|  | Pure error | 0.15 | 12 | 0.01 | 0.34 | 3.26 |
|  | Lack of fit | 0.02 | 4 | 0.00 |  |  |
| Lignin: aerosil | Regression | 37.27 | 1 | 37.27 | 3275.13 | 4.49 |
|  | Residual | 0.18 | 16 | 0.01 |  |  |
|  | Total | 37.46 | 17 |  |  |  |
|  | Pure error | 0.12 | 12 | 0.01 | 1.71 | 3.26 |
|  | Lack of fit | 0.07 | 4 | 0.02 |  |  |

**Table S4.** Cochran’s test for the determination of homogeneity of acetone, gelatin:aerosol, gelatin, lignin:aerosol, and lignin curves (n=3, p=5, and 95% confidence level).

| Curve | Variances | | Cochran’s test | |
| --- | --- | --- | --- | --- |
|  | Average | Higher | Test value | Critical value |
| Acetone | 0.015 | 0.013 | 0.835 | 0.684 |
| Gelatin:aerosil | 0.015 | 0.011 | 0.755 |  |
| Gelatin | 0.057 | 0.025 | 0.439 |  |
| Lignin:aerosil | 0.019 | 0.008 | 0.441 |  |
| Lignin | 0.075 | 0.039 | 0.526 |  |
